# Understanding high endothelial venules: Lessons for cancer immunology

**DOI:** 10.1080/2162402X.2015.1008791

**Published:** 2015-05-07

**Authors:** Ann Ager, Michael J May

**Affiliations:** 1 Infection and Immunity; School of Medicine; Cardiff University; Cardiff, UK; 2 School of Veterinary Medicine; University of Pennsylvania; Philadelphia, PA, USA

**Keywords:** dendritic cells, high endothelial venules, lymphotoxin-β receptor, T cell homing, tumor immunotherapy

## Abstract

High endothelial venules (HEVs) are blood vessels especially adapted for lymphocyte trafficking which are normally found in secondary lymphoid organs such as lymph nodes (LN) and Peyer's patches. It has long been known that HEVs develop in non-lymphoid organs during chronic inflammation driven by autoimmunity, infection or allografts. More recently, HEVs have been observed in solid, vascularized tumors and their presence correlated with reduced tumor size and improved patient outcome. It is proposed that newly formed HEV promote antitumor immunity by recruiting naive lymphocytes into the tumor, thus allowing the local generation of cancerous tissue-destroying lymphocytes. Understanding how HEVs develop and function are therefore important to unravel their role in human cancers. In LN, HEVs develop during embryonic and early post-natal life and are actively maintained by the LN microenvironment. Systemic blockade of lymphotoxin-β receptor leads to HEV de-differentiation, but the LN components that induce HEV differentiation have remained elusive. Recent elegant studies using gene-targeted mice have demonstrated clearly that triggering the lymphotoxin-β receptor in endothelial cells (EC) induces the differentiation of HEV and that CD11c^+^ dendritic cells play a crucial role in this process. It will be important to determine whether lymphotoxin-β receptor-dependent signaling in EC drives the development of HEV during tumorigenesis and which cells have HEV-inducer properties. This may reveal therapeutic approaches to promote HEV neogenesis and determine the impact of newly formed HEV on tumor immunity.

## Abbreviations

EC: endothelial cells
FRC: fibroblast reticular cells
HEC: high endothelial cells
HEV: high endothelial venules
LN: lymph nodes
LPA: lysophosphatidic acid
LT: lymphotoxin
LT-βR: lymphotoxin-β receptor
MAdCAM: mucosal cell adhesion molecule
PNAd: peripheral node addressin
SIP: sphingosine-1-phosphate
TLO: tertiary lymphoid organ
VE-cadherin: vascular endothelial cadherin
VEGF: vascular endothelial growth factor

## Introduction

**Figure 1.**
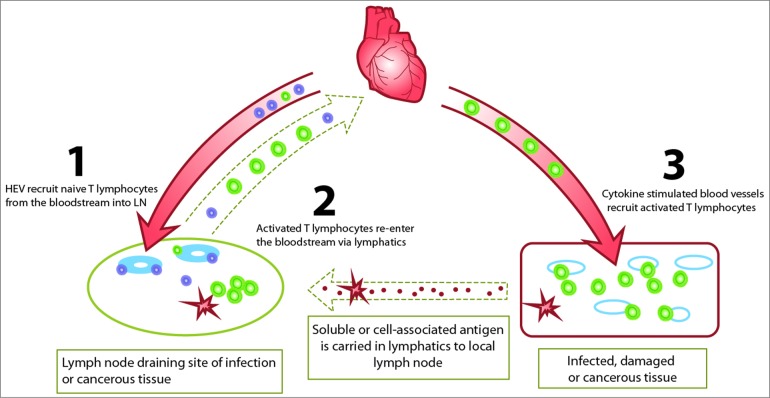
The role of high endothelial venules in T lymphocyte dependent immunity. HEV recruit naive and central memory T lymphocytes from the bloodstream into lymph nodes where they scan antigen loaded dendritic cells that have migrated from infected, damaged or cancerous tissues (**1**). Following activation by antigen, activated T lymphocytes exit the lymph node via lymphatics and re-enter the bloodstream (**2**). Activated T lymphocytes are recruited to sites of inflammation by cytokine-activated blood vessels (which are not HEVs) to clear infected or cancerous tissue (**3**).

The raison d'etre of a LN is to filter lymph that passes through it, sequester incoming antigen and mount an appropriate response, be it activation, tolerance or homeostatic proliferation of lymphocytes. To do this, LN must sample the full repertoire of naive and memory lymphocytes in the body.^1,2^ Specialized blood vessels called HEVs are key players in this process because they extract naive and memory lymphocytes from the bloodstream, regardless of antigen receptor specificity, and deliver them into the node under homeostatic conditions.^3^ Here, lymphocytes scan dendritic cells as well as the supporting fibroblast reticular cell (FRC) network for activating, tolerogenic and homeostatic stimuli.^4-7^ Lymphocytes that do not encounter a cognate antigen leave the node within hours and re-enter the same or a different LN during lymphocyte recirculation, which is a fundamental for effective immunosurveillance.^8^ Following activation and differentiation, precursors of effector T lymphocytes, such as T helper cells and cytotoxic T cells, exit the LN via efferent lymphatics after 2–3 d, re-enter the bloodstream and are recruited to sites of inflammation by cytokine-activated blood vessels (which are not HEVs) to clear infection and repair damaged tissues (**Fig. 1**).

## What are High Endothelial Venules (HEVs)?

**Figure 2.**
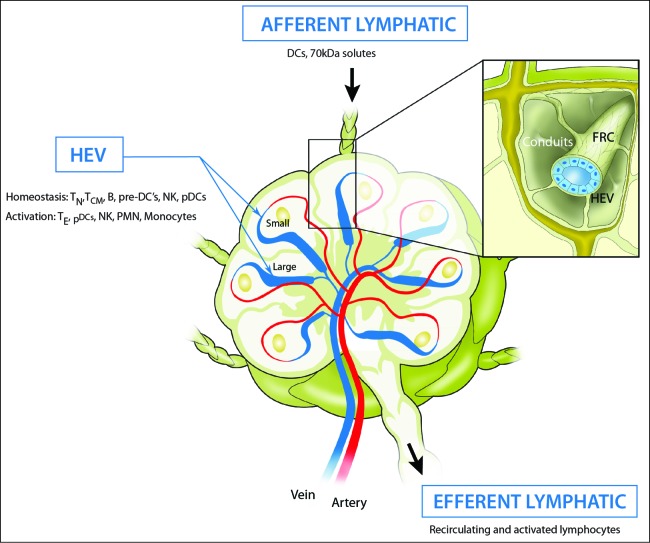
The migration of immune cells in and out of lymph nodes via high endothelial venules and lymphatics. The main artery into the node arborizes into a capillary bed in the outer cortex that leads directly into the post-capillary venular network where HEVs are located. HEVs increase in size as they traverse the paracortex or T cell area of the node and merge with flat-walled venules in the medulla. HEV are ensheathed by fibroblast reticular cells (FRC) that are continuous with the FRC-coated conduits that form the supporting internal scaffold on which lymphocytes and antigen presenting cells crawl during immunosurveillance (insert). Under homeostatic conditions HEV are major portals for entry of naive (T_N_), central memory (T_CM_) T and B cells as well as precursors of conventional dendritic cells (pre-DCs), natural killer (NK) cells and plasmacytoid dendritic cells (pDCs). Effector T cells (T_E_), NK cells, pDCs, neutrophils (PMN) and monocytes can be recruited by HEV in activated LN. Lymphatic vessels form a separate vascular system. Afferent lymphatics drain the surrounding area and deliver tissue-derived dendritic cells (DCs) to the FRC network and <70 kDa solutes to the basal lamina of HEV via the conduit system. Recirculating and activated lymphocytes leave via efferent lymphatics to re-enter the bloodstream.

**Figure 3.**
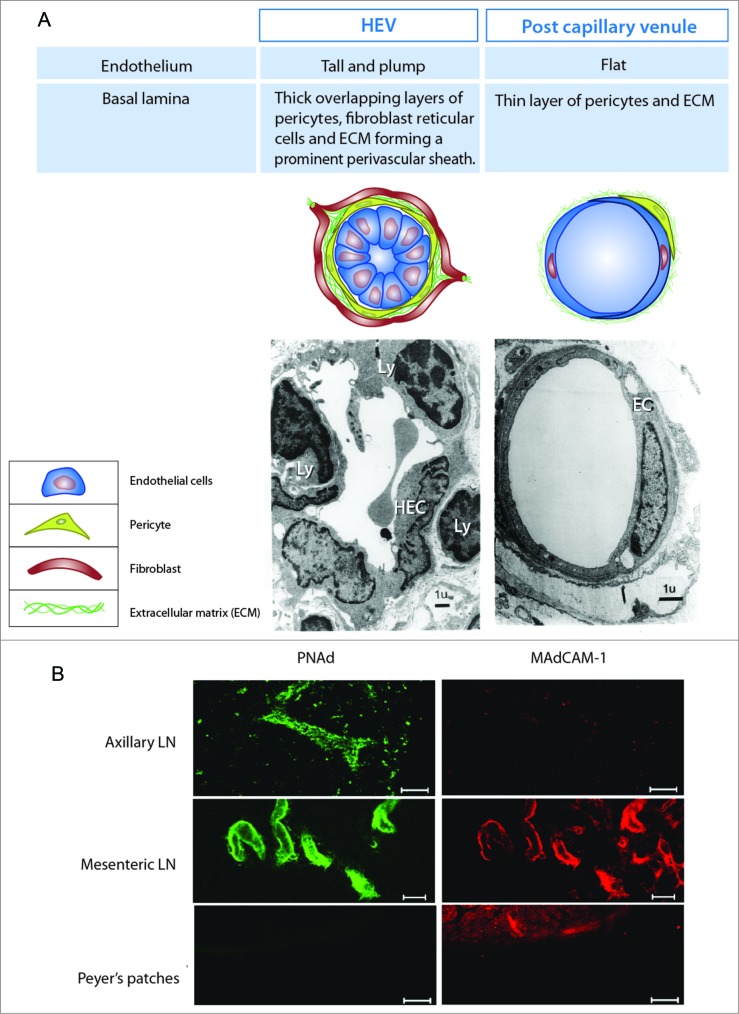
(See previous page). Distinguishing properties of high endothelial venules. (**A**) High endothelial venules (HEV) are lined with plump high endothelial cells (HEC) which contrast with flat endothelial cells (EC) lining non-specialized post-capillary venules. HEC are supported by a thick basal lamina and perivascular sheath of fibroblast reticular cells (FRC). HEV are also characterized by the presence of lymphocytes (Ly) within the endothelial cell lining and basal lamina as shown by transmission electron micrography. (**B**) HEV in subcutaneous (peripheral) lymph nodes of mice such as axillary LN selectively express peripheral LN addressin (PNAd) and HEV in mucosal associated lymphoid organs such as Peyer's patches selectively express the mucosal addressin MAdCAM-1. However HEV in other mucosal associated lymphoid organs such as mesenteric LN co-express PNAd and MAdCAM-1. C57BL/6 mice were injected with anti-PNAd (MECA-79) or anti-MAdCAM-1 (MECA-89) antibody and vibratome sections processed for whole mount immunohistochemistry. Scale bar is 50 μM for LN and 100 μM for Peyer's patches.

HEVs form a branching network of post-capillary venules which is fully integrated into the normal blood vascular bed of all secondary lymphoid organs except the spleen. The HEV network is highly spatially organized, controlling both the site of lymphocyte entry and contributing to the structural organization of LN (**Fig. 2**). Incoming arteries arborize into a capillary bed in the outer cortex or B cell area of the LN and feed directly into the post-capillary venular network where HEVs are found. HEVs gradually increase in size from the smallest at the cortical–paracortical junction and largest vessels in the paracortex or T cell area of the node. HEVs merge with larger flat-walled venules in the medulla which drain into the collecting vein which exits the LN.^9^ A combination of ultrastructural and histochemical studies has been used to identify and characterize HEV within LN. In histological preparations, EC lining HEV have a characteristic cuboidal morphology which distinguishes HEV from other post-capillary venules (**Fig. 3**); it is this morphology that has engendered the name HEVs. Other characteristic features of HEV include a thickened apical glycocalyx and a thickened basal lamina.^10^ The endothelial lining is enveloped in overlapping layers of pericytes and pericyte-like FRC which form a prominent perivascular sheath that is part of the thickened basal lamina.^11,12^

Although a defining feature, the characteristic endothelial morphology on its own cannot be relied on to identify HEV. The height of EC varies significantly between strains of mice, animal species and the method of tissue collection.^10,13,14^ High endothelial cells (HEC) express differentiation markers such as vascular endothelial (VE)-cadherin/CD144 and CD31 that confirm their endothelial identity; however, these markers are expressed by all vascular EC and are not specific for HEC.^15^ A more reliable marker for HEV is the expression of peripheral and/or mucosal addressin (**Fig. 3**). Addressins are expressed on the inner, apical surface of EC lining HEV and are ligands for homing receptors on lymphocytes. Thus, addressins identify the functional capacity of HEV to recruit lymphocytes from the bloodstream into LN.

In adult mice, expression of peripheral node addressin (PNAd), a ligand for L-selectin/CD62L, is a defining feature of HEV since it is not normally expressed by other types of blood vessel inside or outside of lymphoid organs,^16^ although PNAd staining is detected in some activated epithelia.^17^ PNAd expressing HEV are identified by immunohistochemical staining using the rat monoclonal antibody MECA-79 (**Fig. 3**). MECA-79 identifies 6-sulpho sialyl Lewis^x^, (a functional carbohydrate epitope that binds L-selectin) on extended core-1 branched O-linked sugars and detects HEV in human and murine tissues as well as in sheep LN which do not have characteristic high-walled HEV.^10^ The MECA-79 epitope is displayed on a number of serine/threonine-rich mucin domain containing proteins including CD34, GlyCAM-1, podocalyxin, endomucin and nepmucin.^17^ L-selectin also binds 6-sulpho sialyl Lewis^x^ on core-2 branched O-linked sugars as well as N-linked sugars but these are not identified by MECA-79.^18^ Monoclonal antibodies that identify 6-sulpho sialyl Lewis^X^ on both O- and N-linked sugars in mice and humans (including those identified by MECA-79) have recently been described.^19,20^ Interestingly, PNAd is also expressed at the basolateral or ablumenal surface of HEC but its expression is regulated independently of apical PNAd. Basolaterally expressed PNAd has been shown to contribute to lymphocyte homing to LN but its precise role is not fully understood.^17^

The mucosal addressin (MAdCAM-1), a ligand for α4β7 integrin, is used to identify HEV in mucosal lymphoid organs (mesenteric LN and Peyer's patches) of adult mice. However, MAdCAM-1 is not a specific marker of HEV since it is also expressed by blood vessels in the gastro-intestinal lamina propria and the spleen; MAdCAM-1 is also expressed by stromal cells in embryonic LNs.^21^ The serine/threonine-rich mucin domain in murine MAdCAM-1 can be modified with the MECA-79 epitope and bind L-selectin as well as α4β7 integrin^22^; it is not clear if the less conserved mucin domain in human MAdCAM-1 binds L-selectin.^23,24^

The distribution of addressins in human lymphoid organs is similar to that reported in mice. PNAd positive, structurally distinct HEV have been reported in peripheral LN.^25^ MAdCAM-1 is preferentially expressed by HEV in mucosal associated lymphoid tissues, such as the appendix, but is also expressed by non-HEV blood vessels in the lamina propria and submucosa of the gastro-intestinal tract as well as marginal sinus lining cells of the spleen.^25-27^

In adults, PNAd and MAdCAM-1 were originally described to distinguish between HEV in peripheral (subcutaneous) and mucosal LN (mesenteric LN and Peyer's patches). However, expression of these two addressins overlaps in some LN. For example, PNAd is co-expressed by MAdCAM-1 positive HEV in mucosal associated lymphoid organs such as mouse mesenteric LN (**Fig. 3**) and human tonsils^16,25^ and PNAd dominates over MAdCAM-1 in HEV of mucosal lymphoid tissues that develop post-natally, such as nasal- , bronchial- and ocular-associated lymphoid tissues.^28-30^ Addressin expression is also developmentally regulated. PNAd and MAdCAM-1 are co-expressed by peripheral LN HEV *in utero* and neonatally, however PNAd expression is restricted to the baslolateral surface of HEC. During the first weeks of life, MAdCAM-1 expression is downregulated and PNAd is expressed at the apical surface as HEV complete maturation.^31,32^ Addressin expression is also regulated by immune activation; MAdCAM-1 can be re-expressed by peripheral LN HEV and PNAd expression can be downregulated in antigen-reactive LNs of adult mice with consequent changes to the homing properties of HEV.^33,34^

## How Do HEVs Work?

**Figure 4.**
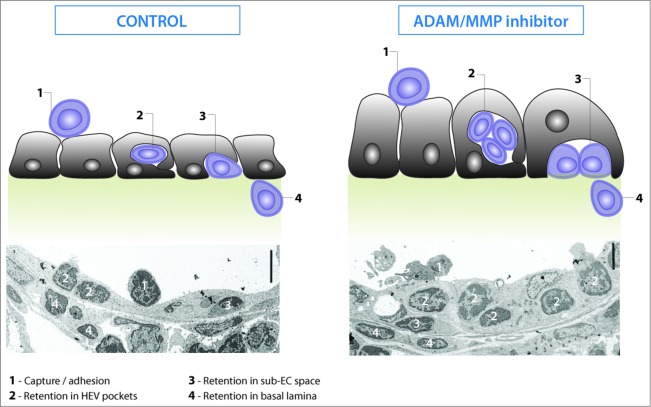
Lymphocyte transmigration across high endothelial venules is a multistage process. High endothelial cells express a molecular address that captures and arrests blood-borne lymphocytes on the inner, luminal surface (**1**). Arrested lymphocytes crawl over the endothelial lining before transmigrating across the wall of HEV. Transmigration can be separated into distinct stages according to the location of migrating lymphocytes. Lymphocytes first transmigrate the endothelial lining where they can accumulate in HEV pockets (**2**). Lymphocytes can be retained in the sub-endothelial space (**3**) before completing diapedesis by crossing the basal lamina and perivascular sheath to enter the LN parenchyma (**4**). Inhibition of ADAM/MMPs arrests lymphocytes within the endothelial lining (stage 2) and the endothelial lining is thickened due to accumulated lymphocytes as shown by transmission electron micrography.^52^

Although widely used to identify HEV, PNAd is only one component of the molecular address required for lymphocytes to home to peripheral LN under homeostatic conditions. The role of apically expressed PNAd is to support the capture and rolling of L-selectin positive blood-borne leucocytes on the endothelial cell lining of HEV. Additional requirements are luminal expression of an arrest chemokine such as CCL21 (or CXCL13 for B cells)^35^ and ICAM-1/CD54, which supports LFA-1 integrin dependent arrest of rolling lymphocytes on the inner, luminal surface of HEV (**Fig. 4**).^36,37^ Naive and central memory T cells as well as B cells are recruited into peripheral LN under homeostatic conditions using this address code. Recent studies have shown that some innate immune cells enter LN under homeostatic conditions using, at least in part, L-selectin and/or CCR7. For example, precursors of classical dendritic cells (pre-DCs),^38^ natural killer (NK) cells^39,40^ and plasmacytoid dendritic cells (pDCs)^41^ have all been shown to enter peripheral LN in unperturbed mice, although in much lower numbers than T and B lymphocytes.

A defining histological feature of HEV is the presence of lymphocytes within the endothelial cell lining and the surrounding basal lamina (**Figs. 3 and 4**)^37^ which suggests that transmigration across the HEV wall is regulated and rate-limiting. This is a complex event involving sequential interactions between migrating immune cells, EC, pericytes, and FRC which is only just starting to be understood. Intravital microscopic analysis of lymphocyte transmigration across HEV has shown that the first step of transendothelial migration from the apical to basolateral endothelial surface takes as little as 3 min.^4^ Although lymphocytes have been reported to penetrate the endothelial cell cytoplasm (transcellular migration) *in vitro*, they also take the conventional route between adjacent EC (paracellular migration).^42^ Lymphocytes take 10 min to migrate across the underlying basal lamina and the surrounding perivascular sheath, however, the close apposition of FRC to the basal lamina of HEV makes it difficult to separate these stages.^36^ Some progress has been made in identifying signaling pathways in lymphocytes that control migration across HEV. Studies using genetically modified mice and pharmacological agents have shown that cooperative signaling downstream of L-selectin and CCR7,^43^ downregulation of cell surface L-selectin by ectodomain proteolysis,^44,45^ affinity regulation of LFA-1^46^ and the intermediate filament vimentin^47^ all regulate transendothelial migration but how these events are integrated is not understood. An additional key regulator of transendothelial migration is lysophosphatidic acid (LPA) which is generated locally by HEV-derived autotaxin and promotes transendothelial migration by inducing polarization and motility in lymphocytes.^48,49^ Lymphocytes must also be able to deform sufficiently to penetrate the HEV wall and this is achieved by contraction of the tail (uropod).^50^ During transmigration, lymphocytes can accumulate apparently within the endothelial lining of HEV in so-called “pockets” but these are extracellular, not intracellular.^51^ The lysophophospholipids LPA and sphingosine-1-phosphate (S1P) may control accumulation in HEV pockets^49,51^ but there are other mechanisms since lymphocytes accumulate within the endothelial lining of HEV in mice treated systemically with a dual metalloproteinase disintegrin (ADAM) and matrix metalloproteinase (MMP) inhibitor (**Fig. 4**), although HEV pockets were not identified in this study.^52^ Identifying the signaling pathways in high and flat EC that control lymphocyte transmigration^53^ may help in unraveling the role of HEV pockets in regulating lymphocyte entry into LN.

Compared to lymphocytes, pre-dendritic cells spend considerably longer within the walls of HEV before entering the LN parenchyma (5 h vs. 10 min for lymphocytes); whether dendritic cells reside in HEV pockets alongside lymphocytes with the potential for cellular cross-talk remains to be determined. What determines residence time within the HEV wall is also an important question to address. Adhesion molecules such as the leucocyte integrins are strong candidates since they switch rapidly between inactive and activate conformations during leucocyte recruitment. For example, VLA-3 integrin at the leading edge of transmigrating leucocytes binding to laminin in the basal lamina and LFA-1 at the trailing edge (uropod) binding to EC regulates retention vs. release of leucocytes in inflamed blood vessels.^54^ Other candidates include activated VLA-4 integrin which binds to fibronectin, another component of the basal lamina.^55^ L-selectin proteolysis may also regulate residence within the walls of HEV^45^ since PNAd is expressed at the basolateral endothelial cell surface^17^ and lymphocytes unable to downregulate L-selectin take longer to transmigrate HEV.^44,52^ The chemokine-rich basal lamina is also likely to control the onward migration of lymphocytes into the LN parenchyma.^43,56^

A unique feature of HEV which is extremely important for controlling lymphocyte recruitment is the connection with afferent lymph. The perivascular FRC sheath that surrounds HEV is continuous with the FRC coated conduit system within LN and forms a communicating unit that delivers incoming lymph-borne soluble factors, such as chemokines and cytokines, directly to the basal lamina of HEV (**Fig. 2**). Button-like attachments between adjacent HEC and reverse transcytosis allow access of chemokines to the luminal surface of HEV where they arrest rolling leucocytes.^57^ The connection with afferent lymph is also important to maintain fully differentiated HEV since expression of PNAd and CCL21 depend on continual stimulation by, as yet, unidentified components in afferent lymph (see below).^33,58-60^

The molecular address on HEC changes dramatically under inflammatory conditions, in part due to the HEV-afferent lymphatic connection which delivers inflammatory mediators from infected or damaged tissues directly to the basolateral surfaces of HEV. *De novo* expression of endothelial E- and P-selectins, increased expression of VCAM-1, presentation of inflammatory chemokines and binding of blood cells or microparticles allows recruitment of blood-borne leucocytes which are normally excluded under homeostatic conditions because they lack L-selectin and/or CCR7.^61^ Interactions between HEV and activated platelets are important to prevent blood loss in inflamed LN by maintaining vascular endothelial cadherin (VE-cadherin) expression on HEC^34^ and HEV bound platelets can also recruit L-selectin negative lymphocytes into LN.^62^ Depending on the infection or inflammatory stimulus, effector T cells,^63^ effector memory T cells,^64^ NK cells ^39,65^ pDCs,^66^ monocytes.^67,68^ and neutrophils^69^ can be recruited by HEV into activated LN and have divergent effects on the immune response. For example, effector T cell recruitment by activated HEV can progress or resolve ongoing immunity depending on whether antigen presenting cells are killed or primed by incoming effector T cells.^63,64^

## HEVs in Cancer

The presence and precise location of tumor-infiltrating lymphocytes, particularly cytotoxic and memory T cells, is a predictor of clinical outcome in several vascularized tumors including colorectal, lung and ovarian cancer.^70-75^ Conventionally it is thought that effector T cells are generated in organized lymphoid tissues, such as draining LN, and recruited to tumor tissue from the bloodstream (**Fig. 1**). However, the recent finding of HEVs in a number of different human cancers is important since it raises the possibility that naive lymphocytes could be recruited into the tumor tissue via these newly formed blood vessels where an appropriate pro-inflammatory environment would allow the generation of cancerous tissue-destroying effector lymphocytes within the tumor tissue, thus avoiding the dilution associated with their redistribution from draining LN via the bloodstream.

PNAd expressing blood vessels with structural features of HEV have been reported in primary tumors of breast, lung and ovary, as well as in melanoma.^76^ The density of HEVs correlated with the extent of T- and B-lymphocyte infiltration of the tumor suggesting that, as in LN, HEVs are entry point for lymphocytes. In a detailed study of resected tumor tissue from 146 primary, invasive, non-metastatic breast cancers, the density of HEVs (number of vessels/tumor area) correlated with the numbers of infiltrating naive, memory and granzyme^+^ CD8^+^ T cells as well as a gene expression profile typical of Tbet, Th1, CD8^+^, and IFNγ^+^ cells.^76^ The clinical impact of HEVs in these patients following surgery for primary breast cancer was analyzed retrospectively and the density of HEVs correlated with disease-free, metastasis-free and overall survival rates for both global and node-positive breast cancers.^76^ In a study of 225 primary melanomas, the density of HEVs correlated strongly with reduced tumor size, expression of naive T- and Th1-associated genes and the presence of DC-LAMP^+^ dendritic cells.^77^ Although relatively small numbers of patients have been analyzed, these clinical data linking development of HEV to improved tumor immunity are supported by experimental studies in mice where the development of PNAd and/or MAdCAM-1 expressing vessels correlates with reduced tumor growth, increased recruitment of naive/central memory T cells and/or local expansion of T cells within the tumor.^78-80^ However, in a mouse model of infection (Helicobacter pylori)-induced carcinogenesis, the development of PNAd expressing HEV preceded adenocarcinoma formation.^81^ The impact of newly formed HEV on tumor outcome will also depend on whether functionally mature dendritic cells are present in sufficient numbers within the tumor tissue to present tumor-derived peptides to naive T cells and induce full T cell activation. Newly formed HEVs may also recruit immunosuppressive cells, such as regulatory T cells, which will limit effective antitumor immunity. It will, therefore, be important to determine the mechanisms underlying the antitumoral effects of HEV reported in breast cancer and melanoma and whether they operate in other types of clinical cancer.

## Tumor Induced HEV in the Absence and Presence of Extra-Lymphoid Structures

It has long been known that PNAd expressing blood vessels develop at sites of chronic inflammation associated with autoimmunity, infection, allergic inflammation, or graft rejection in experimental mice and patients.^17,28,82-84^ These vessels show histological features characteristic of HEV such as cuboidal endothelium and lymphocytes transmigrating the vessel wall.^25^ In addition, they are most often surrounded by dense lymphocytic infiltrates organized into lymph-node like, T- and B-cell areas which are called tertiary lymphoid organs (TLOs) because of their resemblance to secondary lymphoid organs.

**Figure 5.**
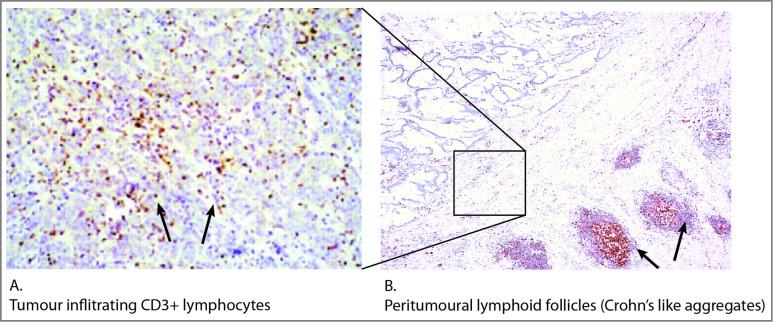
Tumor-infiltrating lymphocytes and tertiary lymphoid organs in colorectal cancer. The location and phenotype of CD3+ lymphocytes infiltrating the tumor tissue has been correlated with patient outcome (**A**).^73^ Lymphocytes are also found in tumor-induced tertiary lymphoid organs/lymphoid follicles in the peritumoral area (**B**). Tumor-infiltrating lymphocytes could be recruited directly from the bloodstream following their activation in draining LN or in peritumoral TLO and release into the circulation, as outlined in **Figure. 1**. Lymphocytes activated in peritumoural TLOs could bypass the bloodstream and migrate directly into the adjacent tumor tissue. Lymphocytes in cryostat sections of tumors were stained either for CD3 (**A**) or mismatch repair enzyme MLH1 (**B**).

TLO have been reported in resected tumor tissues from a range of different cancers,^85^ including non-small cell lung cancer patients,^72^ metastatic melanomas^86^ and breast cancer.^87,88^ In colorectal cancer tissue, tumor-induced lymphoid follicles often containing germinal centers (Crohn's-like aggregates) are found not in the tumor tissue but in the surrounding peritumoral area (**Fig. 5**).^89^ The role of tumor-induced TLOs in regulating tumor progression is just starting to be analyzed. In retrospective studies of lung, breast and colorectal cancer, the presence of these structures is associated with improved patient survival in some studies.^72,90-92^ TLO that support somatic hypermutation and oligoclonal B cell expansion are found in invasive ductal breast carcinomas^93^ and in metastatic, but not primary melanomas,^86^ but the role of locally produced antibodies in tumor progression has not been determined. Experimental studies in mice have highlighted a protumoral, rather than an antitumoral, effect of tumor-induced TLOs. For instance, B16F10 melanoma cells expressing the CCR7 ligand, CCL21, induced the formation of lymphoid tissues at the tumor site, recruited Tregs and myeloid-derived suppressor cells (MDSC) and promoted tumor growth.^94^ The study by Martinet et al.^76^ is the first to measure the density of HEVs in clinical cancers. Although the co-localization of HEV with T- and B-lymphocyte infiltrates was reported, the density of lymphocytic infiltrates or whether they were organized into TLOs was not reported. In a recent study of colorectal cancer, HEV were rarely observed within tumor tissue but were found within organized lymphoid structures in the surrounding peritumoral area.^95^ As reported recently,^96^ the density of HEV containing TLOs did not correlate with improved survival for all stages of colorectal cancer.^95^ The types of immune cell recruited by tumor-derived HEV will be regulated by the local inflammatory environment as well as the organization of stromal cells during the development of TLO. Therefore, the impact of HEV on tumor immunity may change during progression of the disease. It will be interesting to determine whether the antitumoral effects of HEV reported in breast cancer and melanoma are modified by the formation of tumor-induced TLO.

Interestingly, PNAd expressing blood vessels have been reported in tumors in the apparent absence of TLO. For example, PNAd expressing HEV have been reported in primary melanoma in the absence of organized B-cell follicles^86^ and HEVs that form in tumor bearing mice following Treg depletion are not located within highly organized, LN-like T- and B cell infiltrates.^80^ PNAd expressing blood vessels that do not adopt the conventional structure of HEV have also been described in cancer^97^ and interestingly, tumor regression in primary cutaneous melanoma correlated better with the presence of PNAd^+^ vessels lined with flat as opposed to cuboidal EC.^98^ Flat-walled PNAd expressing blood vessels have been observed as early as 3 d following an inflammatory insult^99^ which suggests that HEV development outside of LN can be initiated independently of tertiary lymphoid organogenesis.

## What Drives the Formation of HEV?

In the recent study of primary breast cancer, the density of PNAd expressing blood vessels was associated with longer disease-free survival,^76^ however tumor-induced blood vessel growth is thought to correlate with poor outcome. It is, therefore, important to identify components of the tumor microenvironment that control the development of PNAd expressing blood vessels. Currently, the development of PNAd expressing blood vessels is best understood in mouse LN.

**Figure 6.**
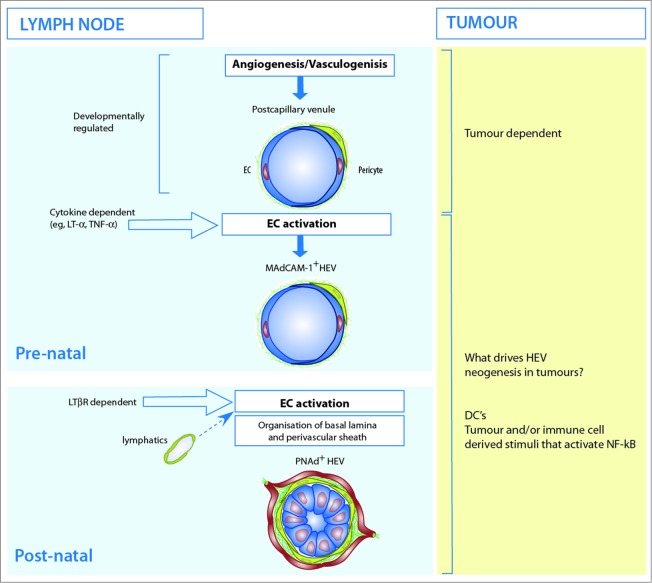
The development of high endothelial venules inside and outside of lymph nodes. **Lymph node**: HEV develop as an integral part of the blood vasculature during embryonic and early post-natal life. Mucosal addressin (MAdCAM-1) is expressed on blood vessel endothelial cells in the late embryo. Luminal expression of peripheral node addressin (PNAd) is induced on MAdCAM-1 expressing blood vessels early in post-natal life and MAdCAM-1 expression is either maintained or downregulated. Engagement of lymphotoxin-β receptor (LT-βR) on endothelial cells drives the development of PNAd expressing HEV. Dendritic cells (DCs) and lymphatics vessels are required to maintain fully differentiated PNAd^+^ HEV and the size of the HEV network is regulated by CCR7^+^ DCs. The stimuli that organize the surrounding basal lamina, perivascular sheath and connecting conduits are not known. **Tumor**: Tumor-derived factors, such as vascular endothelial growth factor, stimulate the growth of new blood vessels to nourish the growing tumor. In mice, tumor-derived ligands for LT-βR stimulate HEV neogenesis and in primary non-invasive breast cancer, dendritic cells (DC) are a candidate HEV-inducer cell since they are a major source of lymphotoxin-β. Whether tumor-derived HEV arise from pre-existing blood vessels during tumor angiogenesis or develop from circulating endothelial progenitor cells during tumor vasculogenesis remains to be determined.

PNAd expressing HEV develop in mouse LN during early neonatal life (**Fig. 6**). Since EC lining HEV are of vascular origin HEV neogenesis may represent differentiation of the LN post-capillary network under the influence of, as yet, unknown factors within the LN microenvironment. Several different approaches have demonstrated clearly that once formed, fully differentiated HEV are actively maintained by an intact stromal compartment. Following isolation of HEC from adult mice, PNAd expression, the distinct endothelial cell morphology and lymphocyte transmigration are downregulated within days,^100^ although rat HEC retain some differentiated properties in culture.^101,102^ Ligation of afferent lymphatics in mice and rats also results in HEV de-differentiation.^58,103^ Administration of a lymphotoxin-β decoy receptor (LTβR-Ig) phenocopies afferent lymphatic ligation in that PNAd expression and HEV function are downregulated in adult mice.^104^ Whether this was a direct effect of blocking LTβR signaling in EC or in other LTβR-expressing stromal cells such as pericytes, FRC or lymphatic EC was not determined.^105-107^

Several recent papers have started to unravel the complex relationship between LTβR signaling and the development of HEV and have highlighted important roles for dendritic cells in the differentiation and growth of HEV. Selective ablation of LTβR expression by vascular EC prevented the development of fully functional, PNAd expressing HEV able to support high levels of lymphocyte trafficking in peripheral LN of mice.^108^ Engagement of endothelial LTβR by CD11c^+^ cells is important in maintaining HEV differentiation since depletion of CD11c^+^ cells results in loss of HEV structure and function in a similar manner to afferent lymphatic ligation.^109^ A separate approach by the Forster lab identified a role for CCR7 expressing CD11c^+^ cells, not in maintaining HEV differentiation, but in regulating the overall size of the HEV network^110^ which suggests that HEV growth and differentiation may be regulated by different types of dendritic cell. Previous studies had shown that tissue-derived dendritic cells stimulate expansion of the HEV network via LTβR dependent release of vascular endothelial growth factor (VEGF)-A from the FRC network.^105,111^

Since the known LTβR ligands are membrane bound, an important question is which LT-αβ expressing dendritic cells make contact with EC to induce and/or maintain HEV differentiation. The study by Moussion and Girard^109^ showed that subcutaneously administered dendritic cells entering via the afferent lymphatics were able to maintain fully differentiated HEV in CD11c^+^ depleted mice.^109^ However, tissue-derived dendritic cells are attached to the FRC network inside LN and have not been reported to make contact with HEC.^112-114^ In contrast, pre-DCs contact HEC during recruitment from the bloodstream and reside within the HEV wall for up to 5 h but whether they express LT-αβ has not been determined.^38^ Studies using mice deficient in different types of dendritic cell will be useful to unravel the impact of dendritic cells and LTβR signaling on the development and growth of HEV.^38,115^ Other LT-αβ expressing cells which are important for LN organogenesis and remodeling should also be considered as HEV-inducer cells, such as RORγt^+^ lymphoid tissue inducer cells and B cells. It is also interesting to consider that the effect of afferent lymph on HEV differentiation may not be to deliver LT-αβ expressing HEV-inducer cells, such as tissue-derived dendritic cells, but rather as a source of chemokines which gain access to HEV via the conduit system where they position HEV-inducer cells alongside HEC to deliver the contact signals necessary for HEV differentiation.

Whether HEV development and growth are driven by dendritic cells and/or LTβR signaling in tumor blood vessel EC in clinical cancer remains to be determined (**Fig. 6**). Evidence in support of this hypothesis comes from a study of primary non-invasive breast cancer patients which showed that DC-LAMP^+^ dendritic cells represent the major source of LT-β in tumor tissues and their presence correlates with the density of HEV. In primary melanoma, the density of HEV also correlated with the presence of DC-LAMP^+^ dendritic cells.^77^ However, as in LN the majority of dendritic cells are localized outside the basal lamina of HEV and very few are in direct contact with HEC.^116^ LTβR dependent HEV neogenesis in seen in experimental animals in which LT-α or LIGHT are directly targeted to tumor cells,^78^ raising the possibility that cells other than dendritic cells could drive HEV neogenesis in cancer. Interestingly, direct intratumoural injection of CCL21 secreting dendritic cells recruited and primed naive tumor reactive T cells within the tumor and resulted in reduced tumor growth. In view of the findings of dendritic cell dependent HEV differentiation, it will be interesting to determine whether the effect of dendritic cells in controlling tumor growth depends on HEV neogenesis.^117,118^

## Can Tumor Blood Vessels Be Manipulated to Promote HEV Dependent Lymphocyte Homing?

If the induction of HEVs in tumor tissue correlates with reduced tumor progression, an obvious goal would be to stimulate HEV neogenesis in tumors but we know very little about the factors that control HEV neogenesis outside of LN. Some clues have come from studies in which cytokines were ectopically expressed in pancreatic islets of mice. Expression of lymphotoxin-α induced MAdCAM-1 but both lymphotoxin-α and lymphotoxin-β were required to induce PNAd expressing blood vessels.^119^ The balance of LT-α vs. LT-αβ expressing cells may therefore drive the development of MAdCAM-1 and PNAd expressing HEV in non-lymphoid tissues.

LT-α and LT-αβ both activate the classical NF-κB pathway characterized by nuclear translocation of p50-RelA complexes. However, LT-αβ  also activates the alternative, non-canonical NF-κB pathway that is hallmarked by NF-κB-inducing Kinase (NIK)-dependent activation of IκB kinase (IKK)-α and nuclear translocation of p52-RelB complexes.^120^ Non-canonical NF-κB signaling is thought to play a dominant role in HEV neogenesis since blockade of LTβR, but not TNFR, downregulates several HEV-specific markers such as GlyCAM-1, MAdCAM-1, CCL21 and HEC-6ST (the sulphotransferase that generates apically expressed PNAd).^104^ In addition, IKKα^(AA)^ mutant mice with defective non-canonical NF-κB signaling lack functional HEV.^28^ Conversely, mice lacking full-length p100 protein which express constitutively active p52, display aberrant PNAd expressing HEV in the spleen.^121^ However, recent studies suggest that there is considerable overlap between classical and non-canonical NFκB signaling in driving the expression of HEV-associated genes.^122,123^ For example, recombinant TNF-α,  LT-α and LT-αβ all induce expression of MAdCAM-1 in EC isolated from human and mouse tissues. HEC-6ST gene expression is induced in EC by soluble recombinant LT-αβ and by TNF-α in human, but not mouse, EC (MJM, unpublished).^124-126^ However, induction of PNAd glycoproteins has not been reported in EC isolated from non-lymphoid tissues, indicating that stimuli in addition to activation of NF-κB signaling are required for HEV neogenesis.

**Figure 7.**
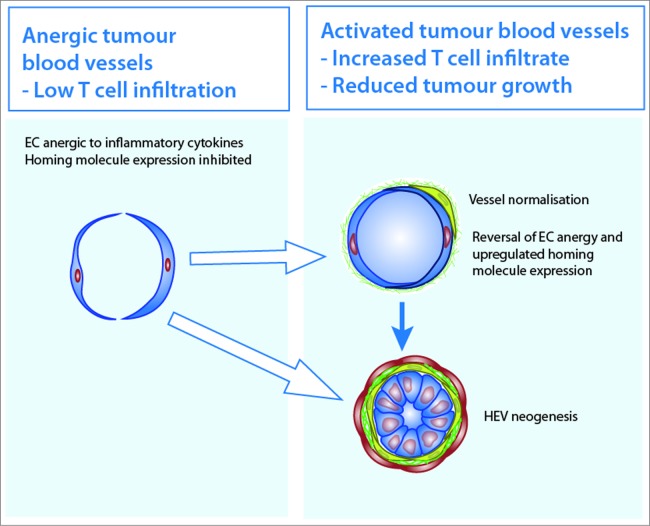
Manipulating tumor blood vessels to promote T lymphocyte homing in cancer immunotherapy. *Left* Tumor blood vessels are anergic to inflammatory cytokines that normally upregulate endothelial cell (EC) expression of homing-associated molecules for T lymphocytes. Tumor-derived factors such as endothelin-B and vascular endothelial growth factor also limit the expression of homing-associated molecules thereby restricting the recruitment of T lymphocytes. *Right* The recruitment of pericytes to immature tumor blood vessels leads to vessel normalization which is associated with increased immune cell infiltration and reduce tumor growth. Vessel normalization reverses EC anergy and upregulates expression of homing-associated molecules which recruit cancer-destroying T lymphocyte. Tumor-derived HEV may recruit naive and central memory lymphocytes and allow the generation of tissue-destroying lymphocytes within the tumor tissue. The development of HEV in tumours may occur independently of vessel normalization.

If HEV neogenesis could be induced how might this impact tumor immunity? Clinical data and experimental studies in mice suggest that the tumor microenvironment restricts the recruitment of cytotoxic, effector T lymphocytes from tumor blood vessels.^127^ This restriction could be considered an immune checkpoint which needs to be overcome for effective immunotherapy (**Fig. 7**). For example, following vaccination or adoptive T cell therapy of tumor-bearing patients, even when tumor-specific T cells comprise 20% of circulating lymphocytes, the outcome on tumor growth can be small.^128^ In mice, tumor blood vessels are anergic to inflammatory cytokines that upregulate CD8^+^ T cell homing in non-involved peritumoral vessels^129^ and tumor-derived factors, such as endothelin-B, suppress T cell recruitment by limiting endothelial expression of homing-associated molecules such as ICAM-1 and VCAM-1.^130^ Endothelial cell anergy may be related to the pro-angiogenic tumor environment; VEGF and FGF prevent cytokine induced homing molecule expression by EC.^131^ Another potential mechanism of limiting cytotoxic T cell entry is the induction of FasL on tumour EC.^132^ The recent finding that Foxp3 expressing Tregs suppress blood vessel differentiation by limiting HEV neogenesis in tumors is yet another strategy by which tumors restrict lymphocyte entry from bloostream.^80^

Different experimental approaches have been explored to increase the recruitment of effector CD8^+^ T cells by tumor blood vessels. Targeted delivery of TNF-α using monoclonal antibodies that bind to tumor vessels promotes T lymphocyte infiltration of tumors indicating that endothelial cell anergy can be overcome.^133^ Expression of ICAM-1 and VCAM-1 can be induced by irradiation^134^ and hyperthermia induced IL-6 trans-signaling leads to increased effector T cell tumor infiltration and a reduction in tumor growth.^129^ Interestingly and somewhat counter-intuitively, anti-angiogenic therapy promotes CD8^+^ T cell infiltration of tumors^131^ and increases the efficacy of adoptive CD8^+^ T cell therapy in experimental mouse models.^135^ This could be a direct consequence of increased homing molecule expression on tumor vessels. However, rather than inhibit tumor angiogenesis, anti-angiogenic therapy has been reported to “normalize” tumor blood vessels by promoting pericyte recruitment and increasing tumor vessel perfusion.^136^ Pericyte maturation also promotes tumor blood vessel normalization and has been shown to increase immune cell infiltration and reduce tumor growth.^137-139^ It is possible that normalized tumor blood vessels recruit more lymphocytes because they can support the shear stresses required to maintain lymphocyte rolling^140^ and transmigration.^141^ The development of HEV could promote antitumor immunity by recruiting naive lymphocytes into the tumor, thus allowing the local generation of cancerous tissue-destroying lymphocytes as shown in mice.^79^ It is also possible that, as in LN, locally produced inflammatory mediators activate HEV to recruit effector cells which counteract the panoply of immunosuppressive cells which are enriched in vascularized tumors.^142^ It will, therefore, be important to determine which populations of immune cells are recruited by tumor-induced HEV to dissect their impact on tumor immunity.

## Summary

The recent reports of HEVs in tumor tissue and a correlation with reduced tumor progression has generated interest in how these specialized blood vessels form and their impact on immune responses to tumors. The rise of cancer immunotherapy has re-focused attention on the tumor vasculature and the necessary role that it plays in recruiting effector lymphocytes able to destroy tumor cells. HEV neogenesis would represent a novel approach to cancer therapy which is diametrically opposed to the long-standing goal to block tumor angiogenesis. However, anti-angiogenesis therapies have not performed as well as first hoped. Although many aspects of HEV biology are still to be unraveled, the recent findings that lymphotoxin-β receptor-dependent signaling in EC is critical for the development and function of HEV are significant advances in our understanding and may provide therapeutic approaches to promote HEV neogenesis in tumors and determine the impact on HEV on tumor immunity.
